# Aerobic Exercise Induces Alternative Splicing of Neurexins in Frontal Cortex

**DOI:** 10.3390/jfmk6020048

**Published:** 2021-05-31

**Authors:** Elisa Innocenzi, Ida Cariati, Emanuela De Domenico, Erika Tiberi, Giovanna D’Arcangelo, Veronica Verdile, Maria Paola Paronetto, Virginia Tancredi, Marco Barchi, Pellegrino Rossi, Claudio Sette, Paola Grimaldi

**Affiliations:** 1 Department of Biomedicine and Prevention, “Tor Vergata” University of Rome, Via Montpellier 1, 00133 Rome, Italy; elisa.inno92@gmail.com (E.I.); emanuela.dedomenico@gmail.com (E.D.D.); marco.barchi@uniroma2.it (M.B.); pellegrino.rossi@med.uniroma2.it (P.R.); 2 Department of Clinical Sciences and Translational Medicine, “Tor Vergata” University of Rome, Via Montpellier 1, 00133 Rome, Italy; ida.cariati@uniroma2.it; 3 Department of Systems Medicine, “Tor Vergata” University of Rome, Via Montpellier 1, 00133 Rome, Italy; erika_tiberi@libero.it (E.T.); giovanna.darcangelo@uniroma2.it (G.D.); 4 Centre of Space Bio-Medicine, “Tor Vergata” University of Rome, Via Montpellier 1, 00133 Rome, Italy; 5 Department of Movement, Human and Health Sciences, University of Rome “Foro Italico”, Via dei Robilant 1, 00135 Rome, Italy; veronica.ver@live.it (V.V.); mariapaola.paronetto@uniroma4.it (M.P.P.); 6 Laboratory of Cellular and Molecular Neurobiology, IRCCS Fondazione Santa Lucia, Via del Fosso di Fiorano 64, 00143 Rome, Italy; claudio.sette@unicatt.it; 7 Department of Neuroscience, Section of Human Anatomy, Catholic University of the Sacred Heart, Largo Francesco Vito 1, 00168 Rome, Italy

**Keywords:** exercise, neurexins, frontal cortex, hippocampus, alternative splicing, synapsis

## Abstract

Aerobic exercise (AE) is known to produce beneficial effects on brain health by improving plasticity, connectivity, and cognitive functions, but the underlying molecular mechanisms are still limited. Neurexins (Nrxns) are a family of presynaptic cell adhesion molecules that are important in synapsis formation and maturation. In vertebrates, three-neurexin genes (NRXN1, NRXN2, and NRXN3) have been identified, each encoding for α and β neurexins, from two independent promoters. Moreover, each *Nrxns* gene (*1–3*) has several alternative exons and produces many splice variants that bind to a large variety of postsynaptic ligands, playing a role in trans-synaptic specification, strength, and plasticity. In this study, we investigated the impact of a continuous progressive (CP) AE program on alternative splicing (AS) of *Nrxns* on two brain regions: frontal cortex (FC) and hippocampus. We showed that exercise promoted *Nrxns1–3* AS at splice site 4 (SS4) both in α and β isoforms, inducing a switch from exon-excluded isoforms (SS4−) to exon-included isoforms (SS4+) in FC but not in hippocampus. Additionally, we showed that the same AE program enhanced the expression level of other genes correlated with synaptic function and plasticity only in FC. Altogether, our findings demonstrated the positive effect of CP AE on FC in inducing molecular changes underlying synaptic plasticity and suggested that FC is possibly a more sensitive structure than hippocampus to show molecular changes.

## 1. Introduction

### 1.1. Beneficial Effects of Aerobic Exercise on Brain Health

AE is known to produce beneficial effects on brain health [1,2,3] and is considered a potent neuroprotective factor [4,5]. In humans, sustained exercise has been clearly demonstrated to enhance learning and memory, improving executive function, and counteracting age-related mental decline [6,7]. Although the effects of physical activity on the brain are relatively widespread, there are specific regions that seem more sensitive than other brain areas. Notably, these regions are strongly associated with executive and cognitive functions and include hippocampus and FC. Previous studies in rodents have shown that exercise stimulates angiogenesis [8], increases the length and number of the dendritic interconnections between neurons [9], and stimulates neurogenesis and the circulation of important neurotrophic factors, such as brain-derived neurotrophic factor (BDNF) [10]. However, the molecular mechanisms underlying the relationship between AE and brain plasticity in different brain areas are still unknown. 

### 1.2. Role of Neurexins in Neuroplasticity

Neuroplasticity refers to the brain’s ability to modify its internal connections in response to external stimuli [11]. Changes in cortical neuron connectivity require modifications of synapse number and strength. At the molecular level, synapsis formation, maturation, and resolution are regulated by a network of adhesion molecules at presynaptic and postsynaptic sites. Among the best characterized molecules, there are NRXNs, which work as important organizers of synapses [12,13,14]. In both mice and humans, NRXNs are encoded by three genes (*Nrxn1, Nrxn2,* and *Nrxn3*), each encoding for a larger alpha (α) and a shorter beta (β) isoform from alternative promoters [15,16]. Further complexity in the NRXN repertoire is achieved through AS regulation of both types of isoforms, thus yielding over thousand different splice variants [17]. AS of pre-mRNAs is ubiquitous in the eukaryotic genome and its importance in brain has recently received a great attention [18,19]. However, the molecular mechanisms that generate splice variants, the potential function of different protein isoforms and what conditions regulate their expression in the nervous system, are not well characterized. All α-NRXNs are alternatively spliced at six sites, referred to as SS 1 to 6 whereas the shorter β-NRXNs contain only SS#4 and 5 [20]. The resulting splice variants can bind a large variety of postsynaptic ligands, playing a role in trans-synaptic specification, plasticity, and strength. AS of the SS4 segment is highly conserved in all *Nrxn* pre-mRNAs and incorporation of the SS4 exon generates SS4+ variants, containing a 30 amino acid insertion, whereas skipping of the SS4 exon results in the SS4− variants. The presence or absence of this exon determines binding of NRXNs to different post-synaptic molecules [21,22,23,24,25,26,27,28], contributing to differentiation, plasticity, specificity of synapses, and behavioral features [29,30]. In vertebrates, NRXNs are synthesized throughout the brain in all excitatory and inhibitory neurons [31,32] and display differential expression patterns, with the abundance of αNrxns exceeding that of βNrxns [31]. Some studies based on single cell RT-PCR and RNA-Seq have revealed cell type-specific Nrxn expression and distinct expression of Nrxn mRNA splice variants in a given cell [17,33,34]. The expressions are modulated by diurnal cycle [35], during development [36], by neurotrophins (NTs) and neuronal activity [37,38], or in response to fear conditioning [39] and to environmental experience such as chronic cocaine treatment [40]. 

The aim of the present study was to investigate the effects of CP AE on molecular changes underlying synaptic plasticity in mouse hippocampus and FC, by studying the alternative splicing of *Nrxn1–3.*

## 2. Materials and Methods

### 2.1. Animal Care and Ethics Statement

All animal breeding, maintenance, and research protocol were performed as described in the project approved by the Ethics Committee of the Interdepartmental Service Centre-Station for Animal Technology (STA)-University of Rome “Tor Vergata” and in accordance with national and international laws and policies (Directive 2010/63/EU of the European Parliament and of the Council, Italian Legislative Decree 26/2014). C57BL/6N mice, provided by the STA (Charles River, Calco, Milan, Italy, RRID:MGI:5656552), were randomly assigned and housed in standard clear plastic cages and kept in light/dark cycle of 12:12 h and ventilation of 10–20 times/h, with ad libitum water and food. Mice were kept in social groups at a constant temperature of 20 ± 2 °C and relative humidity of 50 ± 10%. A block randomization method was used to randomize subjects into groups resulting in equal sample sizes. All efforts were made to minimize the number of animals used and to reduce their suffering. 

### 2.2. Training System and Schedule

**Figure 1:**
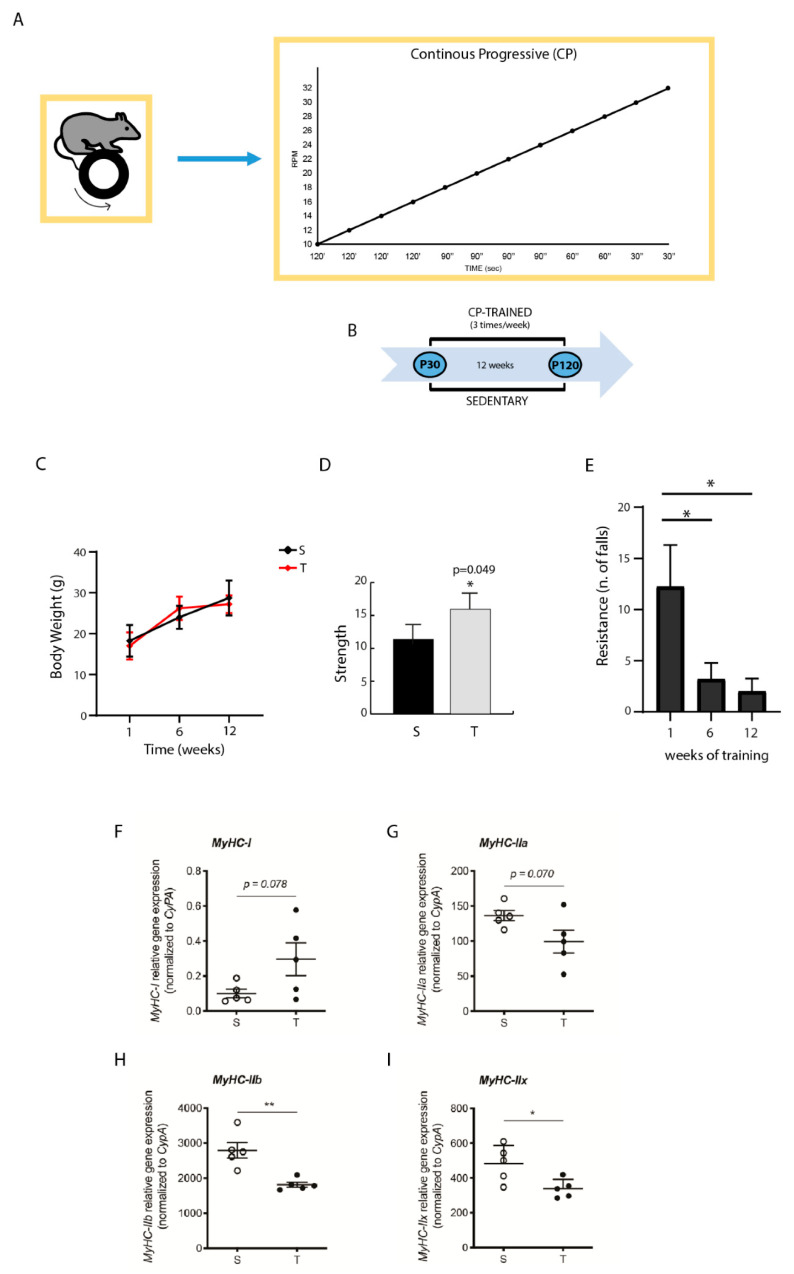
Continuous progressive (CP) aerobic exercise (AE) promotes physical performance and affects muscle fiber expression. (**A**) Schematic representation of CP exercise: CP program was carried out three times a week for a total time of 12 weeks. The training schedule is reported: 120” at 10 RPM, 120” at 12 RPM, 120” at 14 RPM, 120” at 16 RPM, 90” at 18 RPM, 90” at 20 RPM, 90” at 22 RPM, 90” at 24 RPM, 90” at 26 RPM, 60” at 28 RPM, 60” at 30 RPM, and 30” at 32 RPM. (**B**) Timeline of the training program. (**C**) Body weight of sedentary and trained mice at the end of exercise. (**D**) Histogram reporting the strength evaluated at the end of exercise, through a test using a force transducer as reported in materials and methods. (**E**) Histogram reporting resistance as number of falls in trained and sedentary mice. Resistance was evaluated by a test on the RotaRod at constant speed of 10 RPM considering the falls number/time (CP: 1st week 12.3 ± 9.1, 6th week 3.2 ± 3.6, and 12th week 2.0 ± 2.8). * *p* < 0.05. Error bars represent SD. (**F**–**I**) Relative expression of MyHC isoforms of quadriceps muscles from sedentary (S) and trained (T) mice. RT-qPCR analysis shows the expression levels (normalized to CyPA) of MyHC-I, MyHC-IIa, MyHC-IIb, and MyHC-IIx, in S e T mice. Statistical analysis was performed by unpaired Student’s *t*-test and two-way ANOVA (* *p* < 0.05; ** *p* < 0.01).

The new Rotarod 47600 (Ugo Basile, Italy) was used for the training. A total of 10 C57BL/6N male mice were divided into 2 groups: one control sedentary group and one group submitted to CP training program, as previously reported [41,42]. CP program was based on incremental speed changes with gradually increasing intensity of the exercise. It was characterized by three periods: an initial period with a slow rhythm, an intermediate period with a slightly faster pace, and a final period at a fast pace. The speed of the revolutions per minute (RPM) increased gradually starting from a speed of 10 RPM to 32 RPM, without any interruption, with a total of 18 min of training. The mice started CP training at the age of 30 days post-partum (dpp) (P30) and exercise was carried out three times a week for a total of 12 weeks, ending at the age of 120 dpp (P120) (Figure 1A,B). The sample size was arbitrarily set to 10 with 5 animals each, randomly selected.

### 2.3. Animals Assessment 

Animal weight was checked throughout the training protocols (before, during, and at the end of the session). According to conventional procedures [42], all mice were subjected to two different tests to evaluate their physical performance. The strength test was performed using a force transducer (AD Instruments, Ltd., Sydney, Australia; cat. no. MLT050/D). Briefly, the mice were suspended by front limbs while posterior limbs were immobilized. The transduced force was connected to a Lab Chart software program (AD Instruments) for data recording and analysis. The time between the start of the test and the fall was measured. Each mouse performed this test before the training and after the last training session. Sedentary mice performed the same test. Every test consisted in three trials for each animal. Resistance was evaluated using RotaRod at a constant speed of 10 RPM and counting the falls number/time for each training session. The “time limit” was considered as the time between the start of the test and the occurrence of the third fall. If no falls occurred, the test was stopped after 30 min. The first week of exercise of trained mice was characterized by the same number of falls of sedentary mice and, therefore, was considered as a control condition.

### 2.4. RT-PCR Analysis and Quantitative Real-Time PCR

At the end of the training period, all mice were sacrificed according to conventional procedures under anesthesia with halothane (2-Brom-2-chlor-1,1,1-trifluor-ethan). Brains were quickly removed, and hippocampus and FC were dissected and suspended in Triazol reagent (Invitrogen). Similarly, muscle tissues were collected from quadriceps and suspended in Triazol reagent. FC was extracted at different post-natal ages: P30, P75, P120, and P12M (months). Total RNA was extracted from isolated tissues and 1 μg was used for retro transcription (RT) using M-MLV reverse transcriptase (Invitrogen). cDNA was used as template for semiquantitative PCR (RT-PCR) analysis (GoTaq, Promega) or quantitative real-time PCR (qPCR) analysis using PowerUp SYBR Green Master Mix (Applied Biosystems) and Applied Biosystems StepOnePlus Real-Time PCR system (Applied Biosystems) according to the manufacturer’s instructions. All the primers used are listed in Table 1.

### 2.5. Statistical Analysis

GraphPad Prism v 8.4.2 was used to plot all graphs and to perform all statistical analysis. For two-group comparison, unpaired, two-tailed Student’s *t*-test was used to determine the differences between the groups. Ordinary one-way ANOVA (ANalysis Of VAriance) was used to determine the differences between multiple groups; *p* < 0.05 (*), *p* < 0.01 (**), *p* < 0.001 (***), and *p* < 0.0001 (****) were considered to be significant.

## 3. Results

### 3.1. Effect of CP AE on Body Weight, Strength, and Resistance

Mice were trained with CP AE that was characterized by a gradual and progressive increase in intensity, performed three times a week for a total of 12 weeks (see Section 2 and Figure 1A). One-month old mice (P30) were randomly selected and divided in two groups: one group was trained under CP program for 12 weeks, while the corresponding control group was maintained sedentary for 12 weeks (Figure 1B). At the end of training, when mice reached the adult age (P120), physical parameters of trained mice were analyzed and compared to their corresponding sedentary animals. We found that CP training determined an increase in body weight comparable to that of their relative sedentary mice (Figure 1C). In addition, CP training exerted a positive effect on strength, which was significantly increased (Figure 1D), and on resistance, detected as number of falls that were significantly decreased with respect to the sedentary mice (Figure 1E).

### 3.2. CP AE Affects Muscle MyHC Isoform Expression

Skeletal muscle fibers are capable of adjusting their properties to changes in functional demands [45]. This plasticity correlates with the expression of muscle proteins in various isoform combinations and is impacted by changes in neural activity [46]. To analyze whether CP-trained muscles displayed changes in molecular parameters that determine the structural composition of the fibers, we performed qPCR analysis of RNA isolated from quadriceps. Skeletal muscle fibers can be grouped in one type of slow-twitch fibers (type I) and three types of fast-twitch fibers (type IIa, type IIx/d, and type IIb). Type I and type IIa fibers are oxidative, whereas type IIx and type IIb are primarily glycolytic [47]. Remarkably, CP AE caused a significant change in myosin heavy chain (MyHC) isoform expression. Specifically, there was a decrease in the expression of MyHC type IIb (−35%; *p* = 0.0033) and MyHC type IIx (−30%; *p* = 0.0254) glycolytic isoforms (Figure 1H,I), whereas no significant changes were observed in MyHCI and MyHCIIa expression (Figure 1F,G).

### 3.3. CP AE Modulates Neurexin Splicing in FC

**Figure 2:**
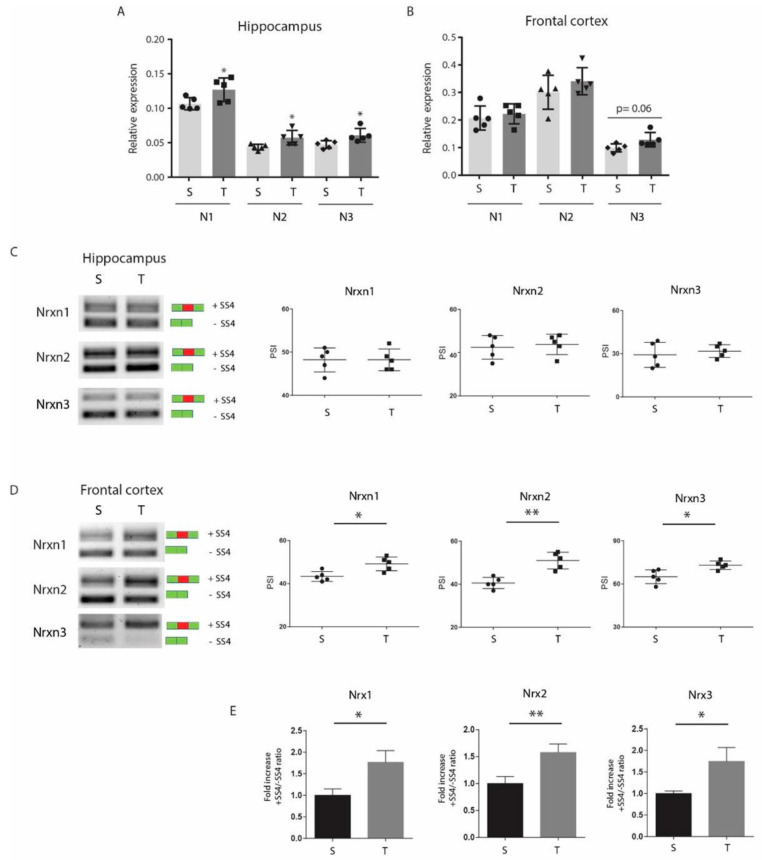
Effect of continuous progressive (CP) exercise on Nrxns splicing in frontal cortex (FC) and hippocampus. Histograms reporting gene expression analysis of total *Nrxns (1–3)* performed by RT-qPCR, using primers for a constant region, on cDNA from RNA extracted from (**A**) hippocampus and (**B**) FC of the sedentary and the CP-trained mice (*n* = 5 per group). β-Actin is used as reference gene. The expression level of *Nrxns* increases in hippocampus but not in FC following CP exercise. RT-PCR of *Nrxns (1–3)* alternative splice variants at SS4 in (**C**) hippocampus and (**D**) FC of the sedentary and the CP-trained mice. Relative scatter plots (in **C**,**D**) show the percent spliced in (PSI) calculated on densitometric values of *n* = 5 mice per group. Densitometric analysis was performed using ImageJ-win64. CP protocol induced a significant increase in +SS4 (inclusion) isoform in all 3 *Nrxns* (Nrxn1 49.20% ± 1.43 in T vs. 43.40% ± 1.03 in S *p = 0.0110*, Nrnx2 51.00% ± 1.63 in T vs. 40.60% ± 1.17 in S *p = 0.0001*, and Nrxn3 73.00% ± 2.17 in T vs. 65.00% ± 1.45 in S *p = 0.0087*). Green box: constitutive splice site. Red box: alternative splice site. Solid circle: sedentary mice. Solid square: trained mice. (**E**) RT-qPCR on cDNA obtained from RNA extracted from FC of the sedentary and the CP-trained mice (*n* = 5 per group). Histograms report PSI as fold change in the trained mice with respect to the sedentary mice. CP protocol increases the +SS4 isoform in all 3 *Nrxns*: Nrxn1 1.77 ± 0.16 in T vs. 1 ± 0.09 in S *p = 0.0131*, Nrxn2 1.59 ± 0.10 in T vs. 1 ± 0.08 in S *p = 0.0091*, and Nrxn3 2 ± 0.17 in T vs. 1 ± 0.04 in S *p = 0.0047*). The expression of +SS4 isoform is normalized with respect to the −SS4 isoform. * *p* < 0.05; ** *p* < 0.01. Error bars represent SD.

We were interested to investigate if CP exercise induced molecular changes in two brain regions: FC and hippocampus. FC and hippocampus from the sedentary and the CP-trained mice were isolated and mRNAs were extracted from them, and the expression of Nrxns was analyzed by qPCR. We showed that CP exercise did not induce any modulation in the expression level of Nrxn genes in FC (Figure 2B), while in hippocampus, a slight and significant increase was detected with respect to the sedentary mice (Figure 2A). Then, using specific primers to detect the splice variants at SS4, we showed that CP exercise induced a significant change of AS of *Nrxn1–3* in FC (Figure 2D), but not in hippocampus (Figure 2C). Notably, CP training promoted the SS4 inclusion in FC, determining an increase in SS4+ in all three Nrxns transcripts with respect to the control sedentary animals (fold increase: 1.13 for *Nrxn1*, 1.26 for *Nrxn2*, and 1.12 for *Nrxn3* vs. sedentary set as 1). The switch from the exon-skipped transcript (SS4−), present in the sedentary mice, to the exon-included transcript, in the trained mice, was clear for *Nrxns 1–2,* but less for *Nrxn3,* since Nrxn3 SS4+ was already the main splice variant expressed in the sedentary animals (Figure 2D). The increase in SS4+ splice variants of all Nrxn genes in FC of the CP-trained mice was further confirmed by qPCR (Figure 2E).

Since *Nrxn1-3* (SS4+) could belong to α or β isoform, we tested which isoform was affected by CP training. RT-PCR analysis using primers specific for either the α or the β isoform indicated that the main increase in SS4+ variants was referred to α isoforms for all *Nrxns* (Figure S1A,B). Nevertheless, a smaller but significant increase in SS4 inclusion in the β isoforms of *Nrxn1* and *Nrxn2* was also detected (Figure S1A,B), whereas *Nrxn3β* was already shifted towards the SS4+ in sedentary mice and was not affected by exercise. Collectively, these results indicate that CP training promoted a shift towards the SS4+ in *Nrxn1-2 α/β* and in *Nrxn3α* isoforms, without affecting *Nrxn1-3* global expression levels in FC.

### 3.4. Neurexin Alternative Splicing in Postnatal Frontal Cortex

**Figure 3:**
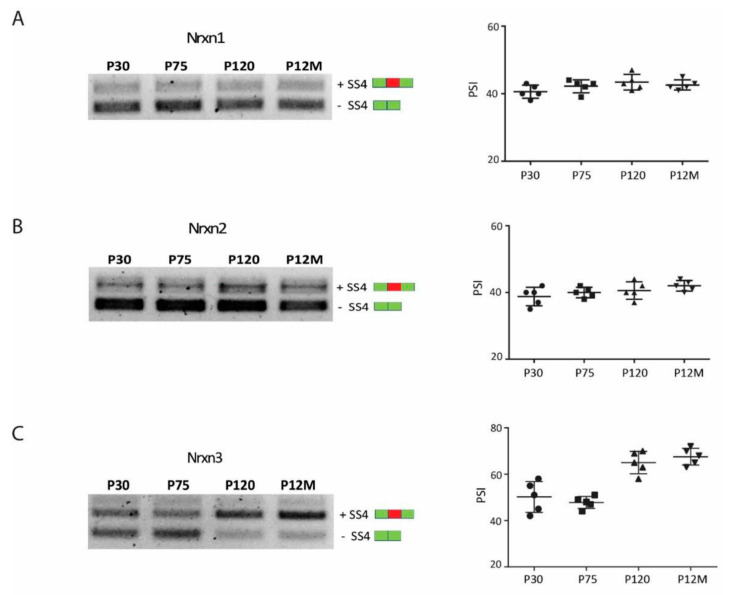
Neurexins splicing pattern in postnatal frontal cortex. RT-PCR analysis of Nrxns splice variants at SS4 in FC of mice at different ages: P30, P75, P120, and P12M (*n* = 5 mice for each group). Representative splicing pattern of Nrxn1 (**A**) Nrxn2 (**B**), and Nrxn3 (**C**). On the right, the corresponding scatter plots shows the densitometric analysis (ImageJ-win64) expressed as PSI (percent spliced in). The splicing pattern for *Nrxn1–2* does not change during age, while *Nrxn3* shows an increase in the +SS4 isoform from adult age. Statistical analysis was performed by ordinary one-way ANOVA. Error bars represent SD. Green box: constitutive splice site. Red box: alternative splice site.

AS is tightly regulated during neurodevelopment, and specific splicing switches accurately define neuronal maturation stages [48,49]. To understand if the expression of *Nrxn1–3* SS4+ splice variants detected in FC in response of CP exercise could be specifically induced by training and not associated to a specific postnatal brain maturation stage, we investigated the splicing pattern of *Nrxn1–3* in FC of mice at different postnatal ages: P30, P75, P120, and P12M. P30 corresponds to the young age when mice started the exercise training, P75 is a young-adult age transition, P120 corresponds to the adult age at which the CP training ended, and P12M corresponds to a more advanced middle-age stage. We found that the main *Nrxn* splice variants expressed at young age (P30) were the skipped exon transcripts (SS4−) for all Nrxn genes (Nrxn1–3) (Figure 3A–C). This pattern was maintained unchanged until middle age for *Nrxn1–2* (Figure 3A,B), while for *Nrxn3,* the expression of the exon including splice isoform (SS4+) upregulated from adult age (P120) (Figure 3C). This finding indicated that AS of *Nrxn1–2* was not modulated during FC maturation, while a change in the expression of *Nrxn3* towards SS4+ splice variant occurred at adult age. However, as previously shown (Figure 2D), a further increase in SS4+ Nrxn3 expression following CP exercise could be detected, suggesting that modulation of AS of all Nrxns induced by exercise was not linked to brain maturation.

### 3.5. CP AE Modulates Expression of Genes Involved in Synaptic Functions in FC

**Figure 4:**
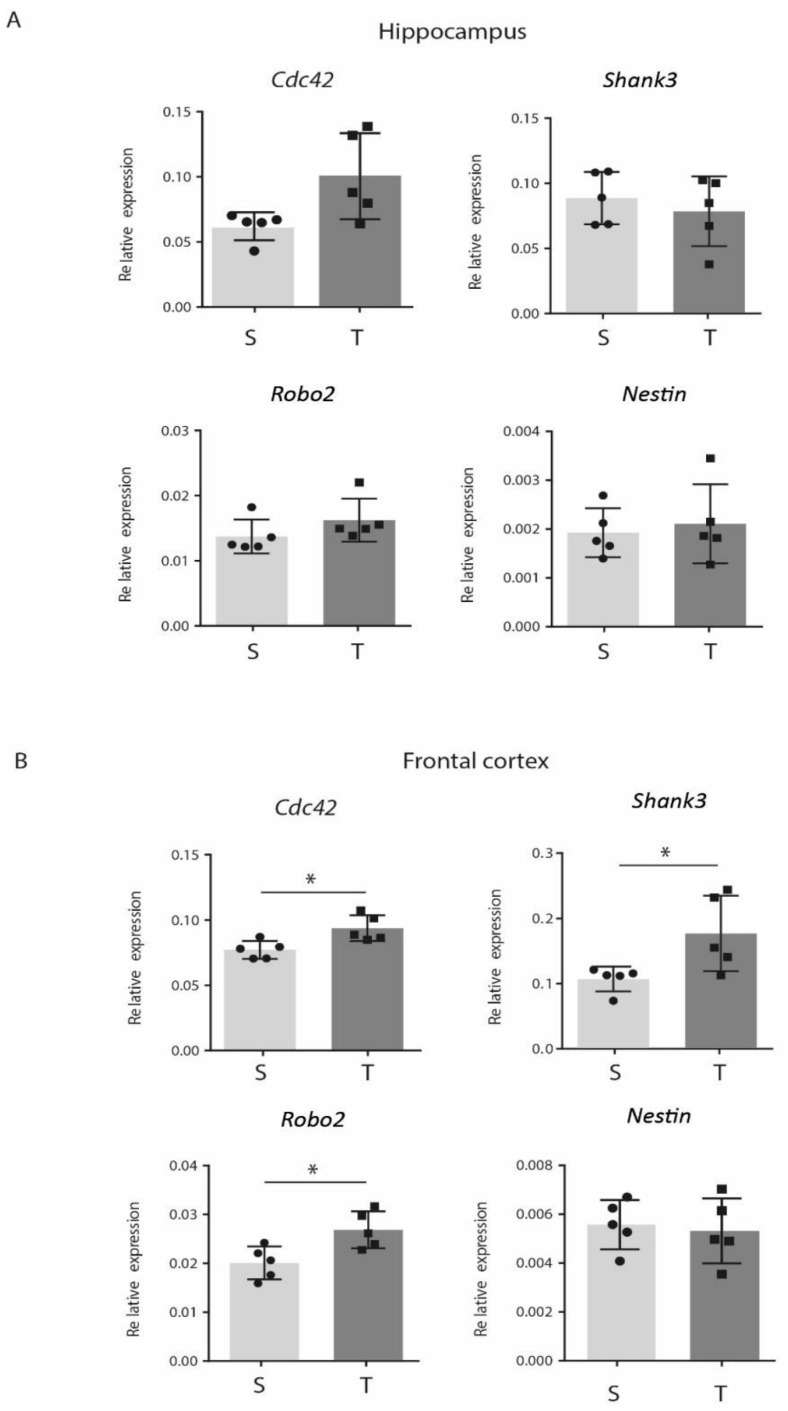
Continuous progressive aerobic exercise (CP AE) modulates gene expression of synaptic-related genes in frontal cortex (FC). Histograms reporting gene expression analysis of Cdc42, Shank3, Robo2, and Nestin analyzed by RT-qPCR on RNA extracted from (**A**) hippocampus and (**B**) FC of the sedentary (*n* = 5) and the CP-trained mice (*n* = 5). CP protocol upregulates (Cdc42 *p* = 0.0153; Shank3 *p* = 0.0417; Robo2 *p* = 0.0177) these genes with respect to the sedentary animals in FC and not in hippocampus. Nestin is not modulated after CP training. β-actin is used as reference gene. (* *p* < 0.05). Error bars represent SD. Solid circle: sedentary mice. Solid square: trained mice.

To evaluate if modulation of Nrxns AS could be part of a global molecular program induced by CP training on FC, we investigated the expression of other genes with well-established roles in synaptic formation, function, and plasticity, namely, cell division control protein42 (*Cdc42*), SH3 and multiple Ankyrin repeat domains3 (*Shank3*), and Roundabout 2 (*Robo2*). CDC42 is a small guanosine triphosphate hydrolase enzyme (GTPase) of the Ras homologous (Rho) family that regulates initial dendritic formation and dendritic spine maturation at both embryonal and adult age. CDC42 protein plays a role in synaptic plasticity, and genetic ablation of the *Cdc42* gene leads to deficits in neuronal polarity and elongation of axons in cultured hippocampal neurons as well as in cortical neurons in vivo [50,51,52,53]. SHANK3 is a synaptic scaffolding protein that forms a key structural part of the post-synaptic density of excitatory synapses [54]. Expression of *Shank3* in a spiny cultured neurons promotes the formation of new synapses, while siRNA-induced knockdown of *Shank3* reduces spine densities and increases spine length [55]. The *Robo2* gene encodes a receptor with axon guidance functions broadly throughout the nervous system [56], playing essential roles in cell migration, synaptogenesis, synaptic plasticity, neuronal survival, and dendritic patterning during prenatal and early postnatal development [56,57,58,59]. By qPCR analysis, we found that expression of all these genes was upregulated in FC with respect to the sedentary controls (Figure 4B) but did not change in hippocampus (Figure 4A). In contrast, the expression of *Nestin*, a protein expressed by neural progenitor cells (NPCs) [60], was modulated neither in FC nor in hippocampus of the CP-trained mice (Figure 4A,B). These results indicated that molecular changes involved in synaptic plasticity, induced by CP exercise, could be detected only in FC.

## 4. Discussion

Several exercise programs have been used to study the beneficial effects on brain, such as wheel exercise and treadmill exercise of low or high intensity, continuous or intermittent, and of short and long duration [61]. In this study, we trained mice with a continuous progressive aerobic exercise (CP AE) program, which has been previously demonstrated to positively impact on muscle tissues [42]. In this study, we investigated the effect of CP exercise on brain molecular changes by studying AS of Nrxns. Although Fang et al. [62] previously showed that treadmill exercise exerted a positive effect on β-Nrxn levels in rat hippocampus; this was the first evidence reporting an effect of exercise on AS of Nrxns. AS is emerged as an important mechanism for the dynamic modification of neuronal functions. Indeed, the expression of specific splice variants can modify signaling properties, synaptic protein functions, and neuronal connectivity [63]. We reported that young mice trained following CP exercise showed increased expression of *Nrxn (1–3)* splice variants that included the alternative exon at SS4. Moreover, exercise significantly increased the SS4+ splice variant of both α and β Nrxn isoforms.

Interestingly, this effect was detected in FC but not in hippocampus, suggesting that FC is possibly a more suitable structure than hippocampus to explore neuronal molecular markers as potential improvement of plasticity. Moreover, in line with other previous data, this result suggests that brain molecular changes induced by exercise are not widespread in the brain but exhibit regional specificity [64,65].

We further demonstrated that the exercise-dependent upregulation of SS4+ variants of *Nrxns1–3* in FC was not correlated to brain maturation stages, since we did not observe any significant modulation of AS of *Nrxn1–2* in the FC of mice from young (P30) to old (P120M) age. As for *Nrxn3* expression, it showed a more variable splicing pattern during brain maturation with an increase in SS4+ variant at adult age (P120).

To understand, if Nrxns AS could be part of a more general molecular program of enhancement of brain plasticity, we analyzed the expression of other genes, Cdc42, Shank3 and Robo2, with well-established roles in the synaptic structure and plasticity. We showed that gene expression levels of these genes were upregulated in the CP-trained mice in FC but not in hippocampus. Thus, we conclude that the expression of Nrxns SS4+ splice variants induced by the CP exercise may represent a molecular change by which neuronal cells could modify their synaptic number and strength through interaction with different postsynaptic ligands, improving FC plasticity.

The physiological significance of alternative splice variants of *Nrxn1–3* has been partially highlighted up to now. The SS4+ splice variants are known to reduce binding affinity to neuroligins (Nlgns) and to preferentially bind to a class of extracellular ligands called cerebellins (*Cbln1–4*) [27,66]. In the hippocampus, the expression of SS4+ splice variants regulates postsynaptic glutamate receptors composition. Dai et al. [67] demonstrated that the SS4+ splice variant of NRXN1 selectively enhanced *N*-methyl-D-aspartate (NMDA) receptor signaling in the hippocampus, exerting a positive effect on plasticity. Since NMDA, one type of glutamate receptor, is expressed at high density both in hippocampus and in the cortex, we speculate that the increased expression of SS4+ variants of *Nrxns1–3* induced by CP exercise may modulate NMDR receptors signaling and enhance synaptic plasticity in the FC. Accordingly, exercise has been reported to induce modulation of NMDA receptor expression, improving brain plasticity [68].

Using a CP training program, we further demonstrated, at molecular level, the efficacy of this training program in inducing muscular transcriptional and functional changes, with a fast-to-slow transition of muscle fiber transcripts that should result in a faster activation of the mitochondrial oxidative metabolism. These results are in line with previous work documenting a switch from glycolytic to oxidative metabolism upon progressive endurance training in mice [69] and in rats [70,71]. It is well accepted that endurance exercise increases skeletal muscle oxidative capacity by stimulating mitochondrial biogenesis and improving their functional parameters [72,73]. The observed decrease in MyHC IIb and MyHC IIx transcripts during endurance exercise in quadriceps shows the transformation of muscle contractile apparatus in accordance with the changes of muscle oxidative capacity. The expression of specific contractile isoforms is a relevant mechanism of regulation of heterogeneity and plasticity of skeletal muscle, reflecting the adaptation of the contractile apparatus to the endurance exercise.

In conclusion, we identified AS of *Nrxns1–3* as a molecular mechanism modulated by exercise, and we suggest that the expression of specific splice variants of NRXNs may represent new molecular markers by which exercise may improve brain connectivity and plasticity.

## Figures and Tables

**Table 1:** List of primers.

**RT-PCR Nrxns**	
**Nrxns SS4 splice variants** [43]	
Nrxn1 (+/−SS4)	Fw: 5′-TGT TGG GAC AGA TGA CAT CGC C-3′
	Rv: 5′-GAG AGC TGG CCC TGG AAG GG-3′
Nrxn2 (+/−SS4)	Fw: 5′-GTG CGC TTT ACT CGA AGT GGT G-3′
	Rv: 5′-CCC ATT GTA GTA GAG GCC GGA C-3′
Nrxn3 (+/−SS4)	Fw: 5′-TTG TGC GCT TCA CCA GGA ATG-3′
	Rv: 5′-AGA GCC CAG AGA GTT GAC CTT G-3′
**Nrxns Alpha/Beta** [44]	
Nrxn1 (+/−SS4)	Fw(α): 5′-CAG CAC AAC CTG CCA AGA-3′
	Fw(β): 5′-CCT GGC CCT GAT CTG GAT AGT-3′
	Rv(αβ): 5′-GAG AGC TGG CCC TGG AAG GG-3′
Nrxn2 (+/−SS4)	Fw(α): 5′-CAC CAC CTG CAC CGA AGA G-3′
	Fw(β): 5′-GTG CCC ATC GCC ATC AA-3′
	Rv(αβ): 5′-CCC ATT GTA GTA GAG GCC GGA C-3′
Nrxn3 (+/−SS4)	Fw(α): 5′-CTG TGA CTGCTC CAT GAC ATC ATATT-3′
	Fw(β): 5′-AAGCACCACTCTGTGCCTATTTCT-3′
	Rv(αβ): 5′-AGA GCC CAG AGA GTT GAC CTT G-3′
**RT-qPCR Nrxns**	
**Nrxns +/−SS4 splice variants:**	
Nrxn1 (+SS4)	Fw: 5′-TAG TTG ATG AAT GGC TAC TCG ACA AA-3′
	Rv: 5′-GAC TCA GTT GTC ATA GAG GAA GGC AC-3′
Nrxn1 (−SS4)	Fw: 5′-GCT ACC CTG CAG GGC GT-3′
	Rv: 5′-GAG GTG GAC ATC TCA GAC TGC AT-3′
Nrxn2 (+SS4)	Fw: 5′-AAT CCC CTA CCG GCT TGG T-3′
	Rv: 5′-CCC CCT ATC TTG ATG GCA GC-3′
Nrxn2 (−SS4)	Fw: 5′-AGA GGT ACC CGG CAG GAC-3′
	Rv: 5′-GAC ACC TGG CCC TGG AAG-3′
Nrxn3 (+SS4)	Fw: 5′-AGG AGT GGC TGC AGG AAA AA-3′
	Rv: 5′-TTG TCC TTT CCT CCG ATG GC-3′
Nrxn3 (−SS4)	Fw: 5′-GAG CAC TAT CCT ACA GGC CG-3′
	Rv: 5′-AGA GTT GAC CTT GGA AGA GAC G-3′
**Nrxns constant region (RC)**	
Nrxn1 (RC)	Fw: 5′-CTGGAGCTGCACATACACCA-3′
	Rv: 5′-TGCCACCACTCCTTGTGAAA-3′
Nrxn2 (RC)	Fw: 5′-GTAGGCTTCAGCACACACCA-3′
	Rv: 5′-TCGTCCGTGCCCACATTAAA-3′
Nrxn3 (RC)	Fw: 5′-CTGGACTTGGCGACTTCCTC-3′
	Rv: 5′-TGAAGCGCACAACGTGGTAT-3′
β-Actin	Fw: 5′-CTG TCG AGT CGC GTC CAC-3′
	Rv: 5′-GCT TTG CAC ATG CCG GAG-3′
**RT-qPCR**	
Cdc42	Fw: 5′-TGCTCTGCCCTCACACAGAAAG-3′
	Rv: 5′-GCGGCTCTTCTTCGGTTCTG-3′
Robo2	Fw: 5′-CGAGCTCCTCCACAGTTTGT-3′
	Rv: 5′-GTAGGTTCTGGCTGCCTTCT-3′
Shank3	Fw: 5′-GCCTTCCCTGCACTCCAATA-3′
	Rv: 5′-GCTTGTGTCCAACCTTCACG-3′
Nestin	Fw: 5′-GCAGGAGAAGCAGGGTCTAC-3′
	Rv: 5′-GAGTTCTCAGCCTCCAGCAG-3′
Mhc I	Fw: 5′-AGGGCGACCTCAACGAGAT-3′
	Rv: 5′-CAGCAGACTCTGGAGGCTCTT-3′
Mhc IIa	Fw: 5′-CCAAGAAAGGTGCCAAGAAG-3′
	Rv: 5′-CGGGAGTCTTGGTTTCATTG-3′
Mhc IIb	Fw: 5′-GCTTGAAAACGAGGTGGAAA-3′
	Rv: 5′-CCTCCTCAGCCTGTCTCTTG-3′
Mhc IIx	Fw: 5′-CGGTGGTGGAAAGAAAGG-3′
	Rv: 5′-CAGGAGTCTTGGTTTCATT-3′
CypA	Fw: 5′-GTCAACCCCACCGTGTTCTT-3′
	Rv: 5′-CTGCTGTCTTTGGGACCTTGT-3′

## Data Availability

The data presented in this study are available on request from the corresponding authors.

## Supplementary Materials

The following are available online at https://www.mdpi.com/article/10.3390/jfmk6020048/s1, Figure S1: CP aerobic exercise promotes +SS4 fragment in alpha/beta *Nrxns* isoforms.
