# Supplementary material for: Aerobic Exercise Induces Alternative Splicing of Neurexins in Frontal Cortex
Source: J Funct Morphol Kinesiol. 2021 May 31;6(2):48. doi: 10.3390/jfmk6020048 (PMC8261640; doi:10.3390/jfmk6020048)
Supplement: Supplementary file 1 [file jfmk-06-00048-s001.zip › jfmk-1211346-supplementary.pdf]

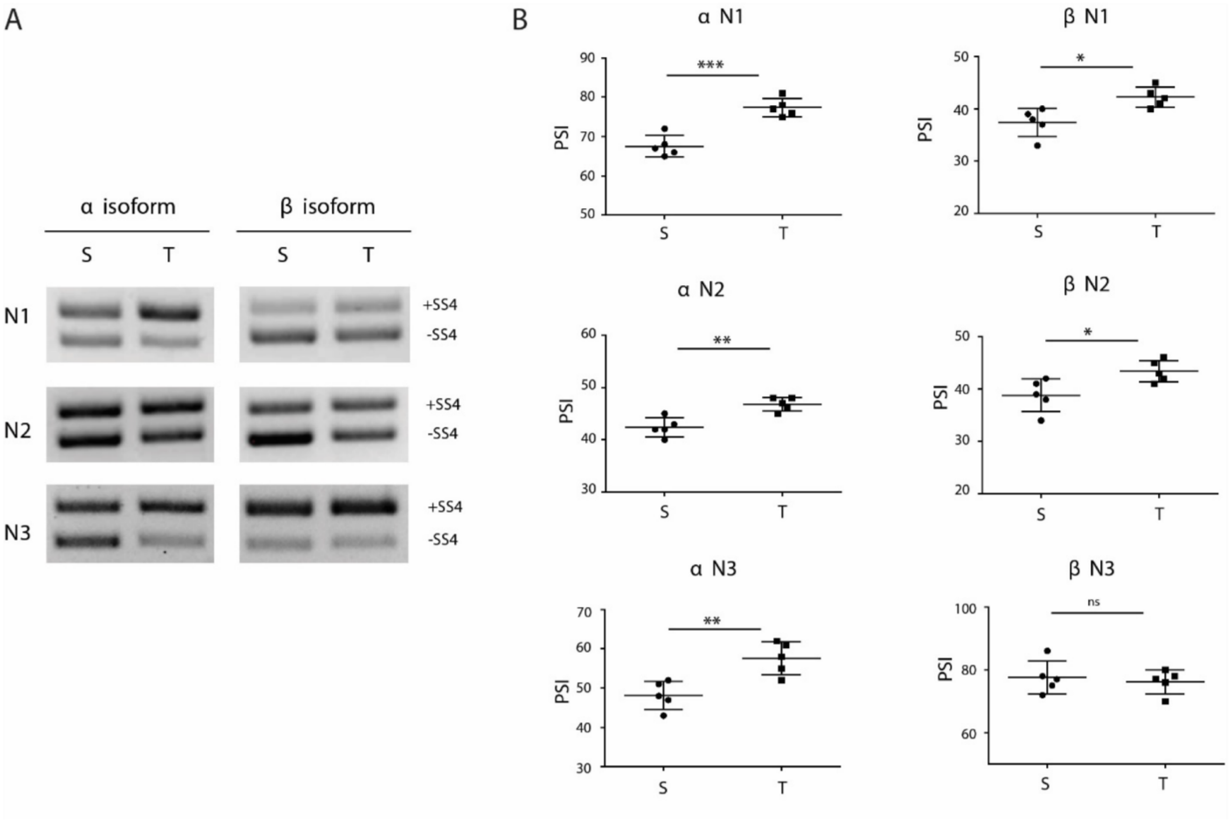

Figure S1. CP aerobic exercise promotes +SS4 fragment in alpha/beta *Nrxns* isoforms. A) RT-PCR analysis showing the representative splicing pattern of α/β Nrxns (1-3) in sedentary mice and after CP protocol. **B**) Densitometric analysis (Image J-win64) is shown as scatter plots of PSI (percent spliced in) for α-Nrxns (α-Nrxn1 77.40% ± 1.03 in T vs 67.60% ± 1.21 in S, p=0.0003; α-Nrxn2 46.80% ± 0.58 in T vs 42.40% ± 0.81 in S, p=0.0023; α-Nrxn3 57.60% ± 1.86 in T vs 48.20% ± 1.59 in S, p=0.0050) and β-Nrxns (β-Nrxn1 42.20% ± 0.86 in T vs 37.40% ± 1.21 in S, p=0.0119; β-Nrxn2 43.40% ± 0.93 in T vs 38.80% ± 1.39 in S, p=0.0251; β-Nrxn3 76.20% ± 1.69 in T vs 77.60% ± 2.34 in S, p=0.6400) *p<0.05; **p<0.01; ***p<0.001. Error bars represent SD.
